# Experiment on the bract stripping and crushing device of a corn harvester

**DOI:** 10.1371/journal.pone.0265814

**Published:** 2022-03-31

**Authors:** Fuxiang Xie, Hongpeng Huo, Xinxin Hou, Yuesheng Fu, Jian Song

**Affiliations:** 1 School of Mechanical-Electronic and Vehicle Engineering, Weifang University, Weifang, China; 2 College of Mechanical and Electronic Engineering, Shandong University of Science and Technology, Qingdao, China; Jamia Millia Islamia A Central University, INDIA

## Abstract

In order to study the mechanism of bract peeling and crushing of a corn harvester, a bract stripping and crushing device was designed that mainly consists of a stripping device, a crushing device, and a frame. The kinematics and dynamics of the roller of bract stripping device were analyzed, and the conditions of bract stripping were obtained. Single factor tests of stripping roller speed and crushing roller speed were carried out. On the basis of single factor tests, orthogonal experiments were carried out to determine crushing roller speed, distance between crushing device axis and stripping device axis, stripping roller speed, and offset angle of stripping roller. The orthogonal experiments showed that the optimum parameters of the device were obtained: the speed of the crushing drum was 1,100 r/min, the axis distance between the stripping and crushing devices was 180 mm, the speed of the stripping roller was 400 r/min, and the offset angle of the stripping roller was 5 degrees. On the basis of orthogonal experiment, three, five, seven, nine and eleven corn feeding and high-speed photography experiments were carried out respectively. The results showed that when three ears of corn were fed at the same time, the effect of corn bract stripping and crushing was the best with the increase of corn number. The stripping roller only grasps most of the bracts of corn at 0.019 s, after stripping the bracts into the crushing device, the bracts were crushed after 0.077 s. The crushing time of bracts was approximately four times as long as that of the bracts.

## Introduction

Corn is an important food crop in China. At present, the harvesting methods of corn harvesters at domestic and foreign mainly include three methods: harvesting the leaves without peeling after harvesting, peeling off the leaves after harvesting, and directly harvesting the grain [1–3]. The current corn harvesting method is mainly the peeling harvesting method after picking ears, which accounts for about 60% of the corn harvesting method [4–6]. The key technology of a corn peeling device has previously been designed and tested [7]. Chai and Lin designed a corn peeling and threshing machine [8, 9], while a corn peeling device for use with a corn combine harvester was designed by Yang [10]. Zhou designed a corn peeling test device [11]. Geng Duanyang et al. established a mathematical model of exciting ear picking with the help of simple harmonic vibration theory, and analyzed the influence law of three main parameters of exciting ear picking model, namely, the diameter of the base circle of ear picking roller, the number of edges of ear picking roller and the rotation speed of ear picking roller, on the ear picking effect. According to the model of exciting picking, the design method of exciting picking roller structure is determined [12]. Shinners et al. designed the model of picking roller in the combine corn harvester, and produced a one-way corn stalk harvester [13–15] and Wang Defu, et al. conducted an experimental study on the crushing process of corn straw, and concluded that the linear velocity at the end of hammer (spindle speed) and the moisture content of corn straw had great influence on the crushing performance of hammer crusher [16]. Furthermore, Petkevichius and Wang analyzed the current status of corn kernel recovery equipment [17, 18], and Hongbin Cong and Hoseinzadeh conducted experimental research on corn stalk laying [19, 20] developed a corn stalk stacking device, using electromechanical-hydraulic integration technology to improve the automation level of the corn harvester stalk stacking device and improve the recovery rate of corn stalks after harvesting.

The combined corn harvesting process is a mechanized process suitable for the corn production system in China. However, there are widespread technical cruxes because it is difficult to separate the corn from the bracts, and the bracts are difficult to smash after separation. One of the main reasons for the high harvest loss rate is the failure of the existing corn peeling device to effectively peel the bract leaves and the injury of fruit ears [21]. The unsatisfactory crushing effect on bract leaves after peeling is the main factor affecting bract leaf recovery and return to the field [22]. However, there are not many studies on the mechanism of bract leaf peeling and crushing. Bract leaf peeling is closely related to the crushing mechanism, the physical and mechanical characteristics of the bract leaf, and the mechanism of the action of the peeling and crushing elements. It is a key technology that needs to be addressed in the research of bract leaf peeling and crushing. In this paper, theoretical modeling analysis, virtual prototype testing, physical prototype testing, and high-speed photography are used to further study the action mechanism and key technology of bract leaf peeling and shredding elements for corn and to develop a optimal corn combine harvester.

### Structural design and working principle

**Fig 1:**
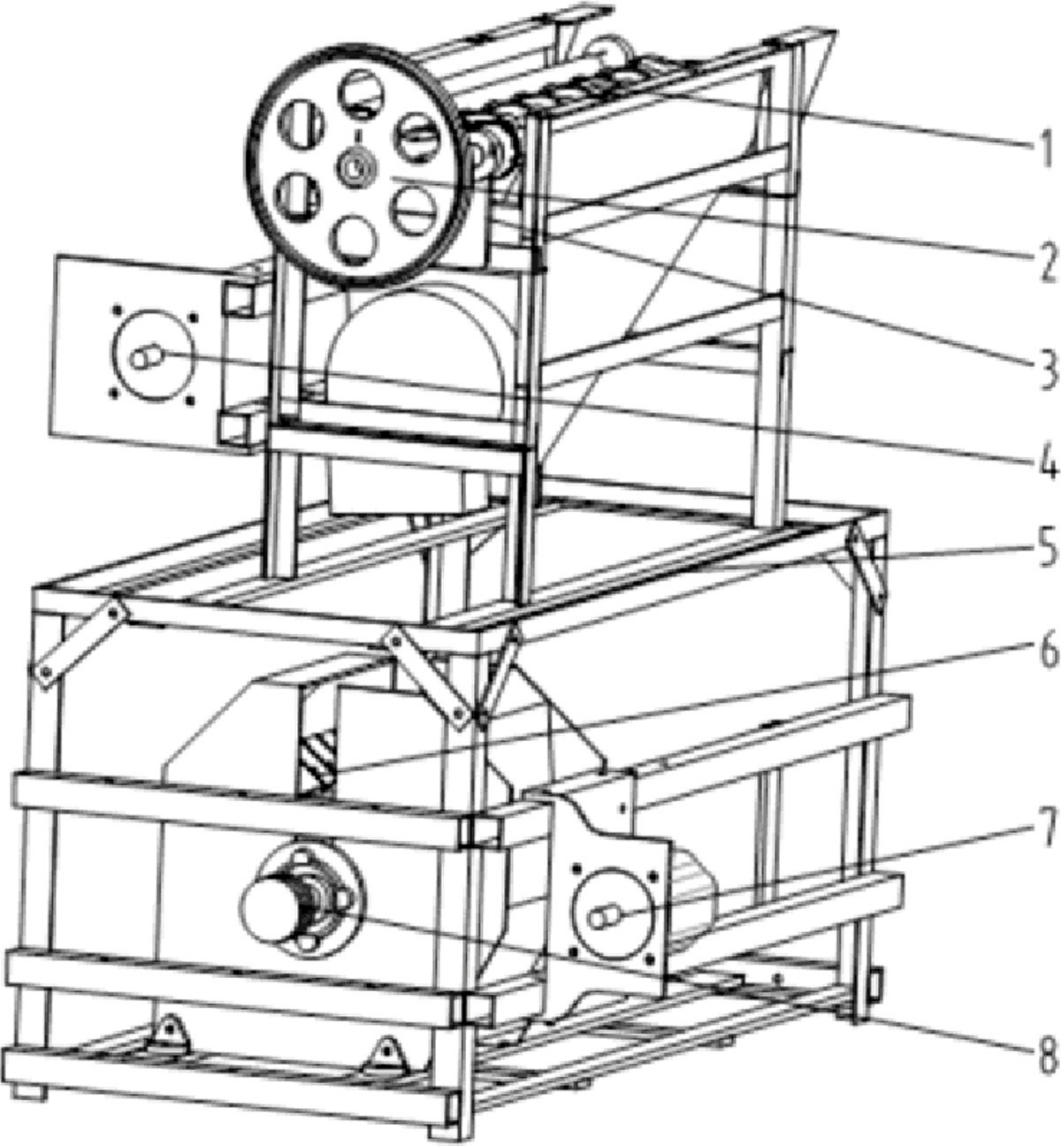
Bract peeling and crushing device of corn harvester. In the picture, 1.Bract leaf peeling rollers 2.Flywheel 3.Transmission mechanism 4.Fixed seat 5.Frame 6.Bract leaf crushing roller 7.Motor 8.Pulley mechanism.

**Fig 2:**
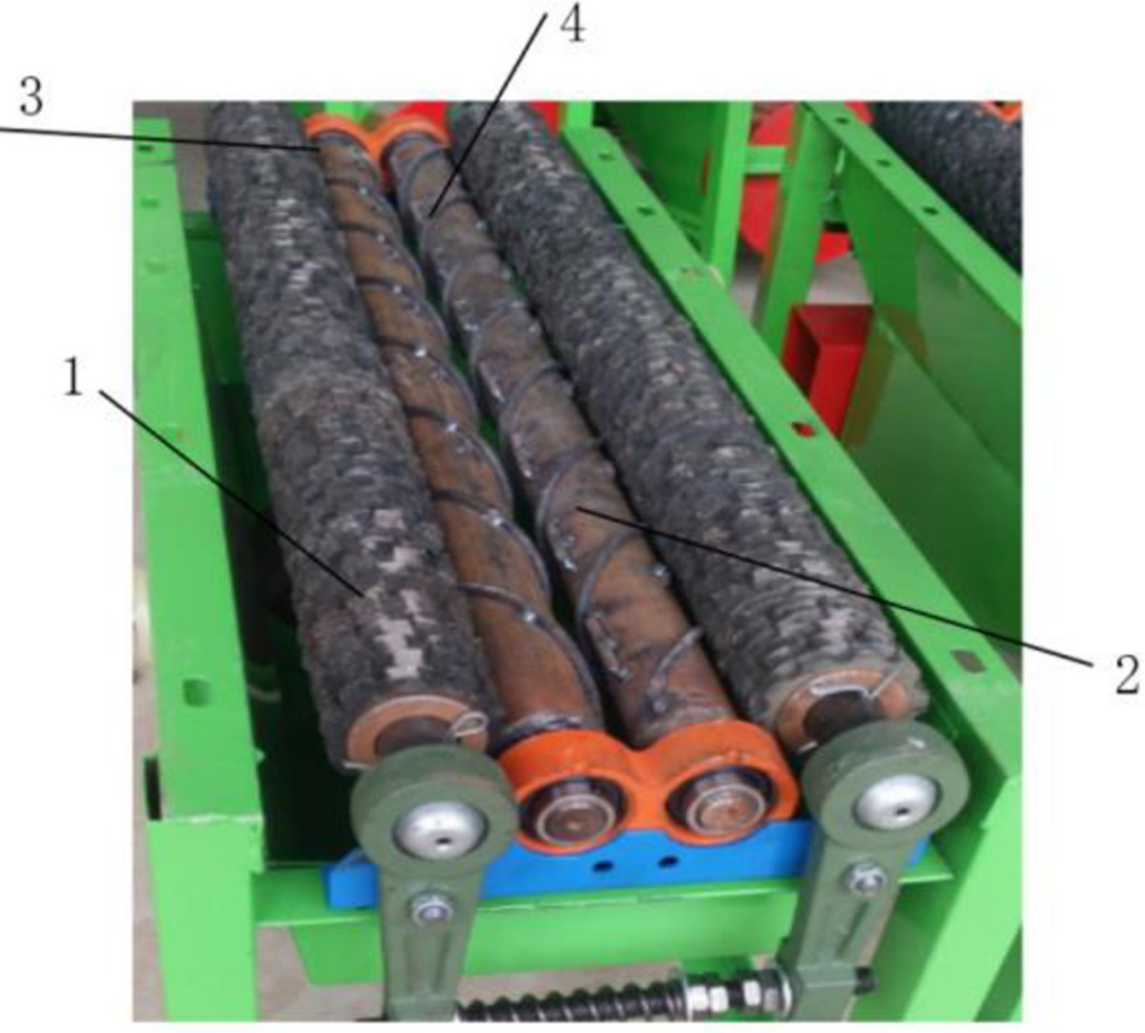
External view of stripping roller. In the picture, 1.The rubber cross 2.The cast steel roll 3.The adjacent rubber cross 4.The winding gap.

A corn bract leaf peeling and pulverizing device is mainly composed of a bract leaf peeling device, a bract leaf pulverizing device, and a frame, as shown in Fig 1. The bract leaf peeling device is mainly composed of a peeling roller, a transmission mechanism, a bract leaf transporting mechanism, and a frame. The external view of the stripping roller is shown in Fig 2. The key to the peeling of corn bracts is the amount of friction between the peeling roller and the corn bracts. The rubber cross flower roller is matched with the cast iron roller, a spiral structure is added outside the cast iron roller, and the adjacent rubber cross flowers are arranged side by side in a staggered discharge. The rubber patch is added to the winding gap, and the surface is provided with increased friction lines. These measures are conducive to increasing friction and improving the effect of bract leaf peeling.

**Fig 3:**
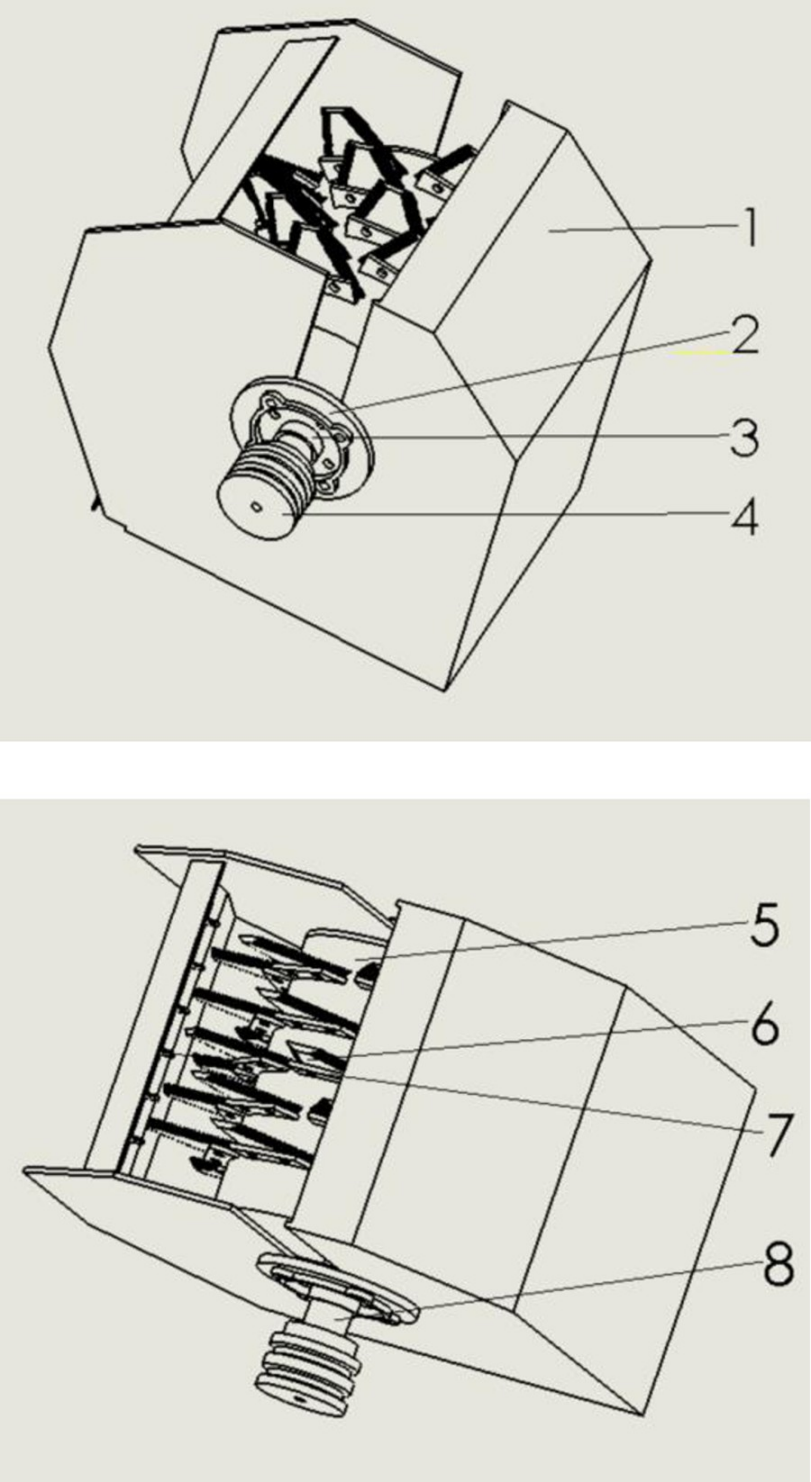
Structure schematic diagram of corn bracts crushing device. In the picture, 1. Crushing box body 2. Flange 3. Bearing seat 4. Pulley 5. Crushing moving blade 6. Crushing fixed blade 7. Crushing roller 8. Transmission shaft.

According to the working process of the corn bud leaf peeling device, a corn bud leaf crushing device was designed. The bract leaf crushing device is mainly composed of a crushing box body, a crushing drum, a crushing moving blade, a crushing fixed blade, a driving shaft, a bearing seat, a chuck, a pulley, and other components. The structure of that corn bud leaf crushing device is shown in Fig 3. The bract leaf crushing device is connected to the transmission shaft through a pulley, the crushing drum is installed on the transmission shaft, and the transmission shaft drives the crushing drum to rotate. The crushing moving blades are distributed across the crushing drum, and the crushing fixed blades are evenly distributed in the front baffle of the crushing box. When the pulverizing drum rotates, the pulverizing fixed blades in the front baffle and the pulverizing moving blades on the pulverizing roller are spatially distributed and cooperate with each other to complete the pulverizing work of the corn bud leaves. After being initially cut, the corn bract leaf fragments can be repeatedly cut by the longitudinal moving blade and the transverse fixed blade under the action of a high-speed rotating pulverizing roller, so the pulverizing efficiency is high. The longitudinal moving blade and the transverse fixed blade are made of low-alloy cutting steel, so the strength and wear resistance of the blade are better. The longitudinal moving blades on the crushing drum adopt a cross-distribution layout, which can effectively crush the corn bud leaves scattered at various positions. The horizontal fixed blades on the inner wall of the box adopt a horizontal uniform distribution method, which is staggered with the moving blades in space and can effectively improve the crushing degree of the corn bracts.

### Force analysis of peeling rollef

**Fig 4:**
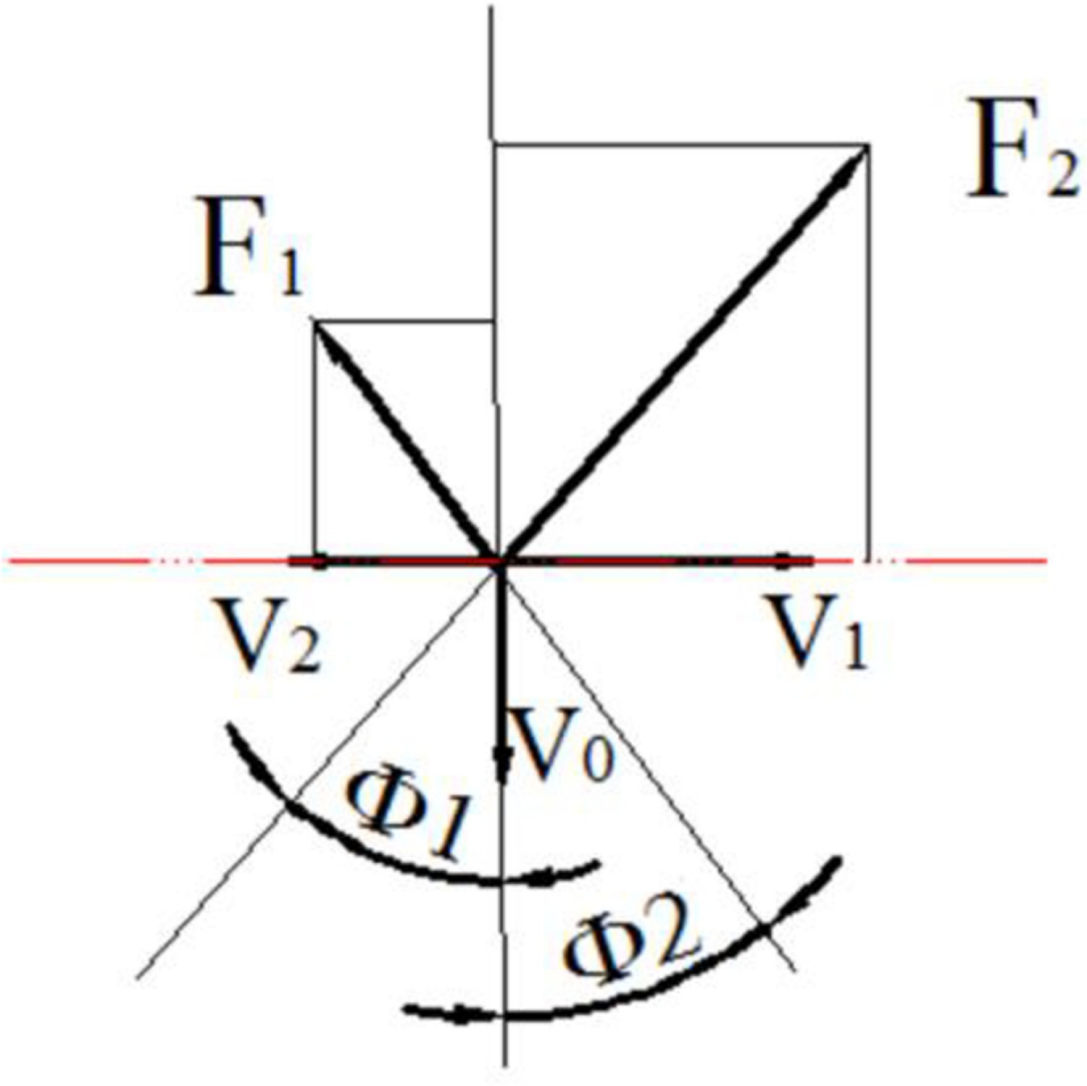
Stripping roller work force analysis. F1 and F2 are frictional forces acting on corn by two rollers, and the instantaneous speeds of the two rollers are opposite. Φ1 and Φ2 are the angle between the friction and the two rollers. V1 and V2 are the linear speeds of corn relative to rubber rollers and cast steel rollers.

**Fig 5:**
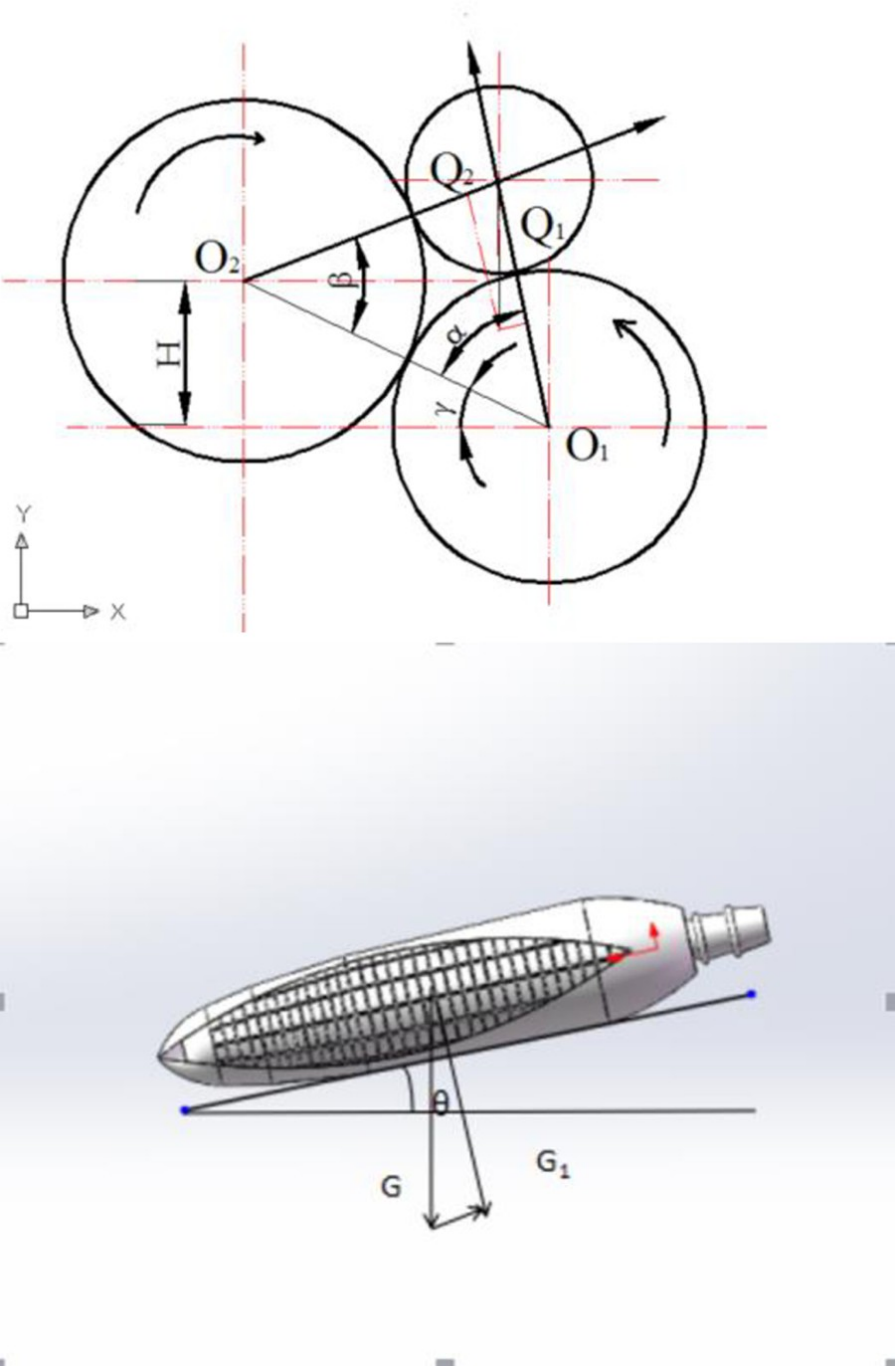
Force of corn when the peeling roller is rotated.

When the peeling roller rotates, the ear not only moves but also rotates. The speed is caused by the action of the rubber roller and the cast iron roller on the ear. It is related to the ratio of roller rotation speed, as shown in Fig 4. Corn moves in two bract leaf peeling rollers; the force is shown in Fig 5.

As can be seen from Figs 4 and 5,the condition formula for corn sliding down the roller:

$$G\;\sin\;\theta >F_{1}\;\cos\;\emptyset_{1}+F_{2}\;\cos\;\emptyset_{2}$$ (1)

Sorted out: *G*_1_ tan *θ*>*F*_2_ cos ∅_2_+*F*_1_ cos ∅_1_

$$\tan\;\theta >f_{1}\frac{\cos\;\emptyset_{1}\;\cos\left(\alpha -\gamma \right)}{\sin\left(\alpha +\beta \right)}+f_{2}\frac{\cos\;\emptyset_{2}\;\cos\left(\alpha +\gamma \right)}{\sin\left(\beta +\alpha \right)}$$ (2)

Where: *G*1 = *Gcosθ*, *N*

When the peeling roller rotates, the corn receives the tangential friction of the roller. At this time, the corn sliding along the roller caused by the tilt is ignored, and the tangential friction is assumed to be F3 and F4.


$$\left\{ \begin{array}{l} F_{3}=f_{3}Q_{1}=f_{3}\frac{G\;\cos\;\theta \;\cos\left(\alpha -\gamma \right)}{\sin\left(\alpha +\beta \right)}\\ F_{4}=f_{4}Q_{2}=f_{4}\frac{G\;\cos\;\theta \;\cos\left(\alpha +\gamma \right)}{\sin\left(\beta +\alpha \right)} \end{array}\right.$$ (3)

Where f3, f4 are the tangential friction coefficients of two peeling rollers on corn when it is rotating.

Because the friction coefficients of the rubber roller and the cast steel roller are different, the corn will rotate in the direction of low friction under the action of the two peeling rollers. The rotation force is as follows:

$$F_{total}=F_{3}-F_{4}$$ (4)

Slewing torque is as follows:

$$M_{0}=F_{total}r=(F_{3}-F_{4})r$$ (5)

When H = 0 and *f*_3_≠*f*_4_,

$$F_{total}=\cos\;\theta \frac{(R_{2}+r){\left(R_{1}+R_{2}\right)}^{2}(f_{3}-f_{4})R_{2}}{\left(\sqrt{{\left(r+R_{2}\right)}^{2}-R_{2}^{2}}R_{1}-R_{2}\sqrt{{\left(r+R_{1}\right)}^{2}-R_{1}^{2}}(r+R_{2})\right)}$$ (6)

When H > 0 and *f*_3_≠*f*_4_,

$$F_{total}={\cos\;\theta \times F}_{A}\times (F_{B}-F_{C})$$ (7)

Of all the above formulas:

Histheheightdifferencebetweenthetwopeelingrollers,N

riscornradius,mm

$${\;R}_{1\;}is\;the\;radius\;of\;the\;rubber\;roller,mm$$

$${\;R}_{2}\;is\;the\;radius\;of\;the\;cast\;steel\;roll,mm$$

αpeelingrollergripangle,°

$$Q_{1}\;corn\;to\;rubber\;roller\;positive\;pressure,N$$

$$Q_{2}\;corn\;on\;cast\;steel\;roller\;positive\;pressure,N$$

$$F_{A}=\frac{\left(R_{2}+r)(R_{1}+R_{2}\right)}{\left(\sqrt{{\left(r+R_{2}\right)}^{2}-R_{2}^{2}}R_{1}-R_{2}\sqrt{{\left(r+R_{1}\right)}^{2}-R_{1}^{2}})(r+R_{2})(R_{1}+r\right)}$$

$$F_{B}=f_{3}R_{2}\sqrt{{\left(R_{1}+R_{2}\right)}^{2}-H^{2}}-\sqrt{{\left(r+R_{2}\right)}^{2}-R_{2}^{2}}H$$

$$F_{C}=f_{4}R_{2}\left(\sqrt{{\left(R_{1}+R_{2}\right)}^{2}-H^{2}}+\sqrt{{\left(r+R_{2}\right)}^{2}-R_{2}^{2}}H\right)$$

$$Q_{1}=\frac{Gcos\;\theta cos(\alpha -\gamma )}{sin(\alpha +\beta )}$$

$$Q_{2}=\frac{Gcos\;\theta cos(\alpha +\gamma )}{sin(\alpha +\beta )}$$

### Successful conditions for bract leaf peeling

#### Bract leaf peeling conditions

The main force of the bract leaf being peeled is the friction between the bract leaf and the peeling roller.


$$f_{1}>f^{\prime },f_{2}>f^{\prime \prime },F_{R}>F_{L}$$ (8)

Where *f*′ is the coefficient of friction between bract leaves and bract leaves;

*f*′′ is the coefficient of friction between bract leaves and corn kernels;

*F*_*R*_ is the pulling force of the peeling roller on the bract leaves, N;

*F*_*L*_ is the tension required to pull off the bract leaves, N.

#### The condition that the bract leaves are not stripped

If the vertical force of the two axes connected to the center line is zero, the bract leaves cannot be peeled off by the roller.


$$F_{1}\;\cos\;\alpha +F_{2}\;\cos\;\beta =N_{2}\;\sin\;\beta +N_{1}\;\sin\;\alpha$$ (9)

Where *N*_1_ = *Q*_1_, *N*_2_ = *Q*_2_, *F*_1_ = *f*_1_*Q*_1_, *F*_2_ = *f*_2_*Q*_2_.


$$Q_{1}(f_{1}\;\cos\;\alpha -\sin\;\alpha )+Q_{2}(f_{2}\;\cos\;\beta -\sin\;\beta )=0$$ (10)

Where *f*_1_ cos *α* = sin *α*, *f*_2_ cos *β* = sin *β*, tan *α* = *f*_1_ and tan *β* = *f*_2_.

Sorted out:

$$\tan\;\alpha =\frac{\sqrt{{\left(r+R_{2}\right)}^{2}-R_{2}^{2}}}{R_{2}},\tan\;\beta =\frac{\sqrt{{\left(r+R_{1}\right)}^{2}-R_{1}^{2}}}{R_{1}}$$ (11)

Because $\cos\;\alpha =\frac{R_{2}}{\left(R_{2}+r\right)},\cos\;\beta =\frac{R_{1}}{\left(R_{1}+r\right)}$.

So in order to prevent that smallest corn ear from bee damaged by extrusion.The diameter of the selected peeling roller should meet the following requirements:

$$R_{2}=\frac{\cos\;\alpha }{1-\cos\;\alpha }r_{min},R_{1}=\frac{\cos\;\beta }{1-\cos\;\beta }r_{min}$$ (12)

### Working principle

During work, the ears are put into the feeding inlet and evenly enter the device where they are distributed between two pairs of relatively peeling rollers. When an ear is rotating on the peeling roller and advancing along the axis of the peeling roller, the bract leaves are continuously rolled by two pairs of relatively moving peeling rollers and peeled by the special peeling roller, and the peeled bract leaves fall through the two peeling rollers into the leaf crushing device. At the same time, under the rotation of the peeling roller, the corn is subjected to the frictional force of the peeling roller to rotate parallel to the rotation axis of the roller. Due to the dual effects of tangential friction and the inclination angle of the peeling roller, the ear moves along the axis to the exit. After, when the corn bract leaves are fed into the crushing device through the corn bract leaf peeling device. Firstly, the corn bract leaves were cut for the first time by a high-speed, vertical, moving blade, and the cut corn bracts are cut by the vertical and horizontal smashing moving blades and the horizontal crushing fixed blades to complete the second cut of the corn bracts. In this way, after the corn bract leaves are cut twice by the longitudinal moving blade and the transverse fixed blade, the crushing process is completed. The turning torque of the bract leaf crushing device is provided by a servo motor, and the torque is transmitted to the bract leaf crushing device through a V-belt drive. The pulverizing roller drives the longitudinal pulverizing blade to run at a high speed. After the corn bract leaves enter the feeding inlet, the longitudinal moving blade and the horizontal fixed blade are staggered to complete the pulverizing work of the corn bract. The crushed corn bract leaf debris is discharged from the discharge port of the corn bract leaf crushing device.

### Test

#### Test equipment

**Fig 6:**
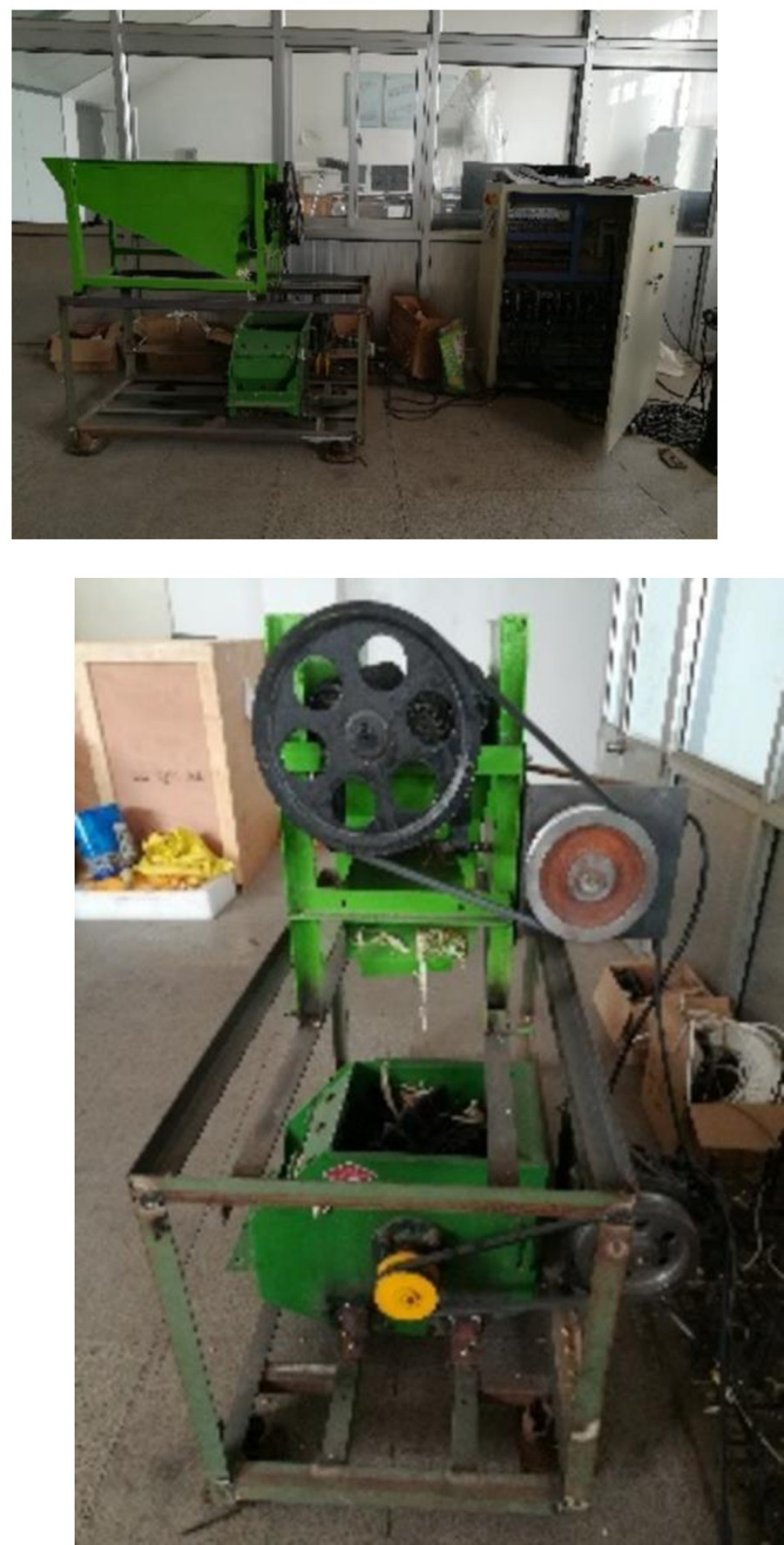
Test bench.

The experiment was carried out in the intelligent engineering laboratory of vegetable planting equipment in Shandong Province. A belt pulley was mounted on the top of the driving roller of the peeling roller, and the other end of the belt was connected to the belt pulley of the motor. The motor was a 110 series servo motor, model 110ST-M05030, with 1.5 KW power, 220 V voltage, and 2,000 rad/min speed. The servo motor controller controls the motor speed to control the speed of the peeling roller. Other test auxiliary equipment included a speed tester (DT2234C, Shanghai Shuangxu), digital camera (Cannon, Japan,Used to obtain and record the whole process of peeling and crushing corn bracts by the test device, so that the running state of the test equipment at different times can be obtained more clearly. See Table 1 for the specific important parameters of the camera.), electronic scale (SL-219, Shanghai Zhejiang Eagle, accuracy 0.01 g), and balance scale (Ohaus, United States, accuracy 0.0001 g) [Fig 6].

**Table 1:** Important parameters of camera.

Parameter name	And numerical range.
Lens classification	Single Lens Reflex
Filter size	72mm
Focusing method	M/A and M
Focal length range	24-85mm

#### Test materials

The test materials used were local mature corn ears. The corn ears used in the test were full and free of diseases and pests to facilitate the most accurate data possible. The moisture content of the test material is shown in Table 2 [23].

**Table 2:** Bracts and kernels dry and wet measurements.

Name		Moisture content								Mean /%
Corn grain	25.6		27	18.4	22.1	24.3	18.8	19.7	23.2	22.4
bract leaf	23		22.2	25	21.4	18.2	25	20	27.3	22.8

#### Test indicators

In the bract leaf peeling and pulverizing device, the test indexes of the device are peeling rate, breakage rate, loss rate, and crushing pass rate [24, 25].

The breakage rate refers to the ratio of mechanical damage and breakage of corn grains to the total mass of corn (without corn cob) peeled off during the leaf peeling process.


$$\eta_{1}=\frac{m_{1}}{M}\times 100\%$$ (13)

In the formula, M is the total mass of the peeled corn with bracts in kg, m_1_ is the mass of the corn kernels with mechanical damage and breakage during the peeling process in kg, and η_1_ is the breakage rate in %.

The peeling rate is the ratio of the weight of the corn with the bracts peeled cleanly, or no more than three bracts to the total weight of the corn with the bracts peeled.


$$\eta_{2}=\frac{m_{2}}{M}\times 100\%$$ (14)

In the formula, M is the total mass of the corn from which the bracts are stripped in kg, m_2_ is the weight of the corn with the bracts stripped, or not more than three bracts, in kg, and η_2_ is the peeling rate in %.

The loss rate is the ratio of the weight of the kernels that fell off during the peeling of the leaves to the total mass of the corn before peeling.


$$\eta_{3}=\frac{m_{3}}{M}\times 100\%$$ (15)

In the formula, M is the total mass of the corn that participated in the peeling of the bract leaf in kg, m_3_ is the weight of the corn shedding kernels during the peeling of the bract leaf in kg, and η_3_ is the loss rate in %.

The crushing qualified rate is the ratio of the number of corn bract leaves to the total number of bract leaves after the width of the corn bract strip was ≤ 10 mm.


$$\eta_{3}=\frac{n}{N}\times 100\%$$ (16)

In the formula, N is the total number of corn bracts and leaves after crushing, n is the number of corn bracts and leaves that meet the standard, and η4 is the crushing qualified rate in %.

### Experiment design

#### Single factor test

The rotation speed of the bract leaf peeling roller and the rotation speed of the pulverizing drum are selected as test factors. The test levels are shown in Table 3. One corn ear was used for each experiment, and each group of experiments was repeated 10 times. The average value was taken. The single factor test was intended to prepare for the orthogonal experiment and provide a reasonable data range for the orthogonal experiment.

**Table 3:** Single factors and levels.

Factor	Standard (r/min)					
Speed of peeling roller	300	350	400	450	500	550
Crushing drum speed	500	700	900	1100	1300	1500

#### Orthogonal test

Four factors, such as the rotation speed of the pulverizing drum, the distance between the axis of the pulverizing and the peeling device, the rotating speed of the peeling roller, and the peeling offset angle, were selected as the experimental factors of the orthogonal test. The factor and level design are shown in Table 4. The orthogonal test (4 factors, 3 layers) was used to design the test, and the L27 (3^13^) orthogonal test table was selected.

**Table 4:** Orthogonal test factors and layers synthesis.

layers			Factor	
	A	B	C	D
1	900	380	350	5
2	1100	280	400	10
3	1300	180	450	15

A is the rotation speed of the crushing drum, and the unit is r/min. B is the distance between the peeling and the axis of the crushing device, and the unit is mm. C is the rotation speed of the peeling roller, and the unit is r/min. D is the peeling offset angle, and the unit is angle.

On the test bench of the corn bract leaf peeling and pulverizing device, the working condition of the corn harvester was simulated, and the peeling and pulverization of the corn bract leaf was studied to analyze which factors played a major role in the bract leaf peeling and pulverizing effect. Each experiment put one ear of corn on the central axis dof the corn bract leaf peeling device, that is, the middle position of the two pairs of peeling rollers of the corn bract leaf peeling device. First, the bract leaves of the ear of corn were peeled off by a corn bract stripping device, and the stripped corn bract leaves were directly dropped into the entrance of the crushing device. The corn bract leaves were then crushed by the crushing device. In order to make the test results more convincing and practical, and it is convenient to count the ratio of test indexes. Reduce calculation error and calculation difficulty.Each group of experiments was repeated 10 times, and the average value was taken.

## Test results and analysis

### Single factor test

**Fig 7:**
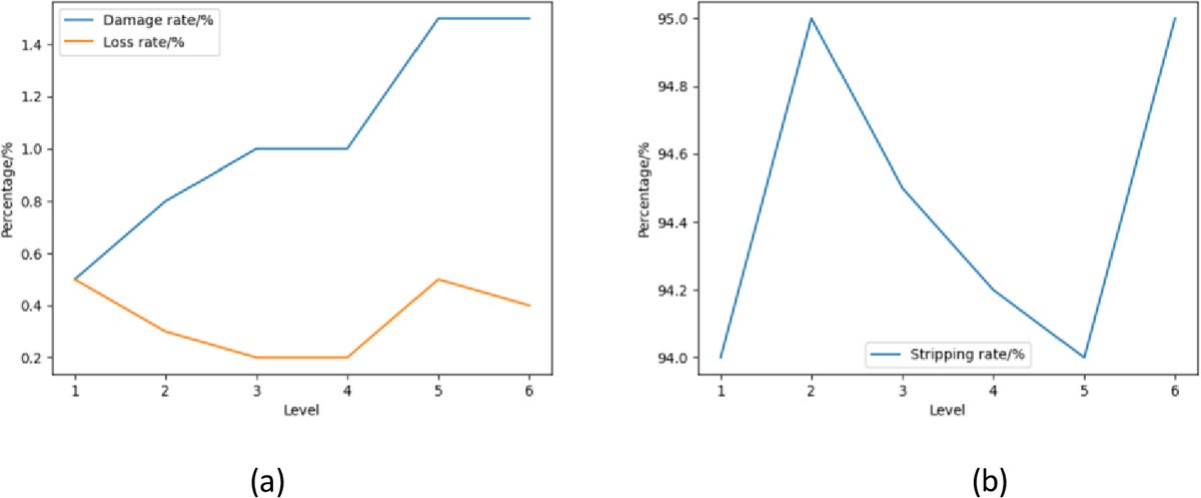
Single factor test results of stripping roll speed.

From the results of the single factor test of the rotation speed of the peeling roller in Fig 7, it can be seen that the various performances of the leaf peeling device increase as the rotation speed of the peeling roller increases. Based on this, the appropriate rotation speed of the peeling roller can be determined. At a speed of 300 r/min, although the damage rate and loss rate are low, the stripping rate is low.When the rotating speed of the peeling roller is 500r/min and 550r/min, the peeling rate reaches 94% and 96%, but at this time, it can be seen that the breakage rate reaches 1% and the loss rate reaches 1.5%. It caused great damage to the ears of corn.Therefore, in the orthogonal experiment, the three speeds of 350 r/min, 400 r/min, and 450 r/min were selected for the speed of the peeling roller. The data obtained from the analysis of variance in Table 5 were compared with the test statistics F table: *F*_0.01_(5,54) = 3.377, *F*_0.05_(5,54) = 2.386. When α = 0.01 or α = 0.05, the F value is greater than the critical value of F, so the speed of the peeling roller of the corn husk peeling device has a significant influence on the peeling effect of the corn husk.

**Table 5:** The single factor analysis of variance table.

Source of variance		Sum of squared variance	Degrees of freedom	Mean square	F	
Factor A	stripping rate	22	5	5.5		1.5
	loss rate	3.5	5	0.7		1
	damage rate	9	5	1.8		4.6
Error	stripping rate	204.75	54	3.7		
	loss rate	40.39	54	0.7		
	damage rate	21.06	54	0.39		

**Fig 8:**
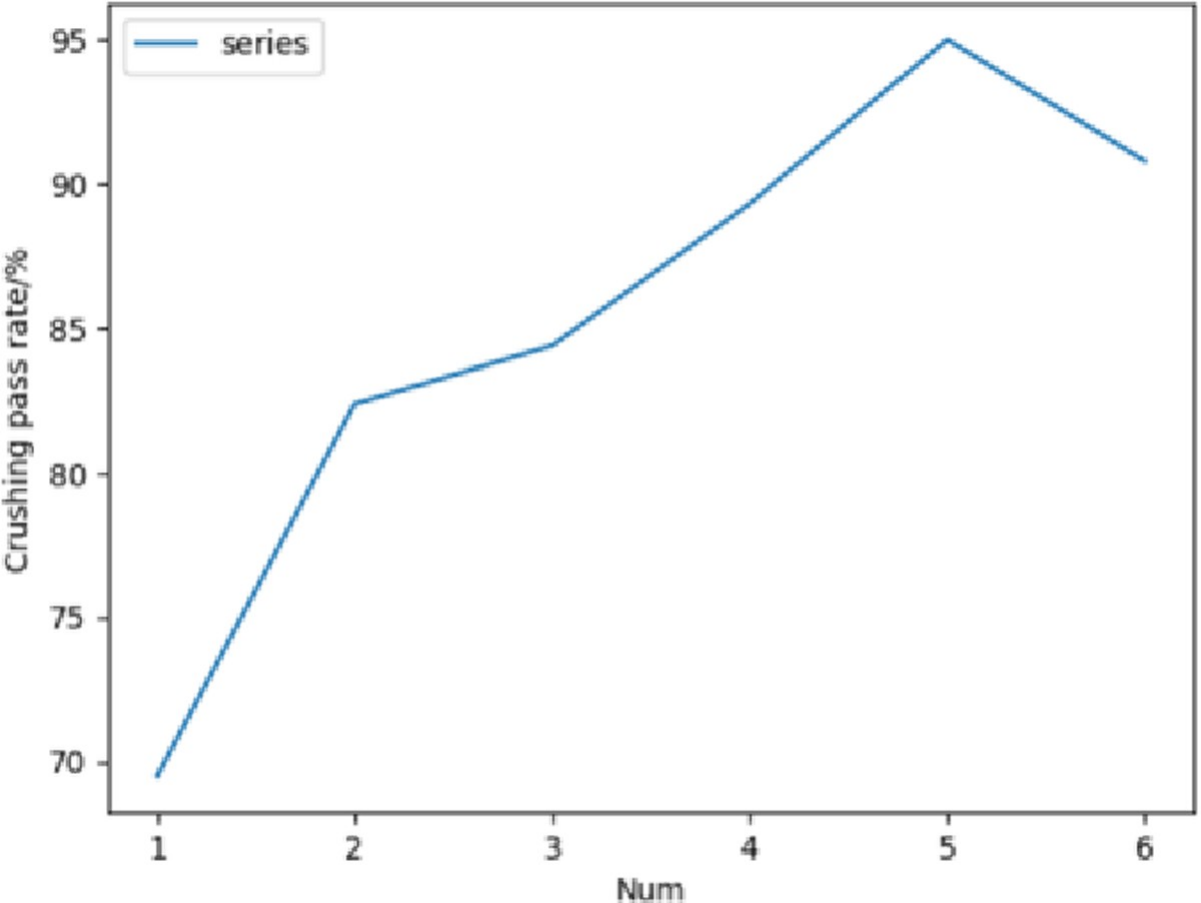
Single factor test results on rotational speed of crushing drum.

According to the single-factor test results of the rotating speed of the crushing roller of Fig 8, it can be concluded that when the rotating speed of the grinding drum reaches the range of 500r/min-1300r/min, with the increasing of the rotating speed of the grinding drum, the success rate of grinding corn bracts also gradually increases, but when the rotating speed of the grinding drum increases to more than 1300r/min, the success rate of grinding corn bracts begins to decline. When the rotating speed is 1300r/min, the success rate of corn bract is the highest, and the grinding effect of corn bract is the best, followed by the rotating speeds of 900r/min and 1100r/min with better grinding effect.

According to the analysis of variance of the single-factor test of Table 6, it can be concluded that at \alpha = 0.01; the F value is greater than the critical value of F, so the rotation speed of the pulverizing roller of the corn bract leaf crushing device has a significant effect on the crushing effect of corn bract leaf.

**Table 6:** Results of variance analysis of single factor test.

	Sum of squared variance		Degrees of freedom	Mean square	F-value	Fa	
Variance squared sum between groups		3915	5	783	74.6		*F*_0.01_(1.5) = 16.26
Sum of squares of variation within the group		567.36	54	10.5			
Sum of squares of total variation		4482.36	59	76			

### Orthogonal test

**Fig 9:**
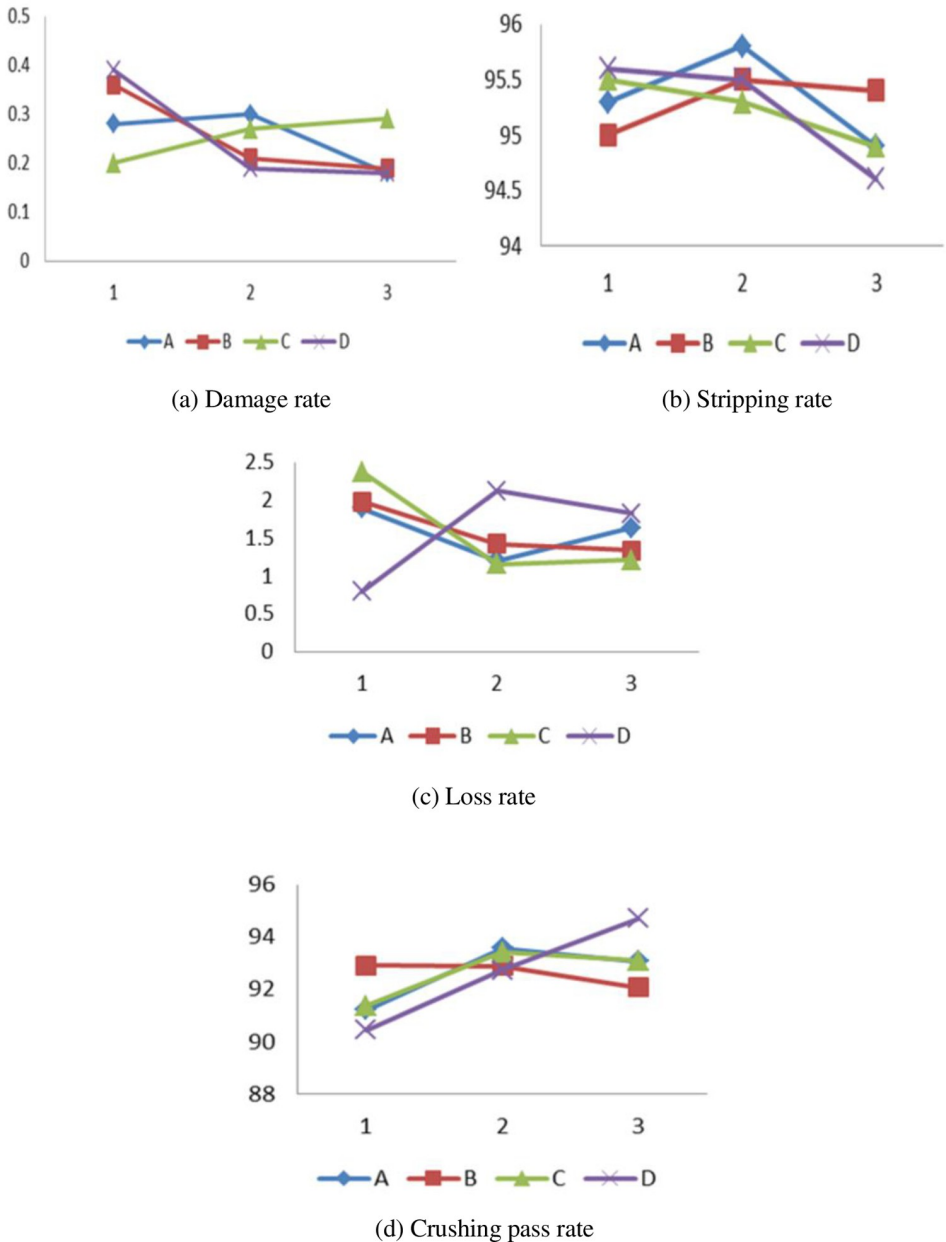
The relation between test factors and test indicators.

The orthogonal test results are shown in Table 7. Of the four test indicators, the larger the peeling rate η2 and the crushing pass rate η4, the better and the smaller the breakage rate η1 and the better the loss rate η3. The optimal parameter combination is analyzed by the comprehensive balance method, mainly considering η2 and η4, followed by η1 and η3. The relationship between orthogonal test factors and test indexes is shown in Fig 9. According to the analysis of variance of the orthogonal test in Fig 9 and Table 8, it can be known that the best combination that affects the damage rate of the test index is A3B3C1D3, and the primary and secondary relationships that affect the damage rate of the test factors are D>B>A>C. It is known from the test that the peeling device plays a major role in the breakage rate. The offset angle of the peeling roller has the greatest influence on the breakage rate. Too small an angle will increase the stress of the corn ear, which will cause the ear to break. The larger the offset angle, the less stress the fruit ear has, and the lower the damage rate. The best combination that affects the stripping rate of the test index is A2B2C1D1, and the primary and secondary relationships that affect the stripping rate are D>A>C>B. The test shows that the factor that has a greater effect on the stripping rate is mainly the offset angle of the stripping roller. The impact of the rotation speed of the crushing drum on the peeling rate is also significant. The larger the rotation speed of the crushing drum, the greater the rotational inertia, and the worse the stability of the test device. This affects the offset angle of the peeling roller. The best combination that affects the test index loss rate is A2B3C2D1, and the primary and secondary relationships that affect the loss rate are D>C>B>A. The test shows that the offset angle of the peeling roller and the rotation speed of the peeling roller are the main factors affecting the stalk rate. Too small of a peeling roll offset angle and larger peeling roll rotation speed will lead to a higher loss rate, and the peeling roll has a greater effect on the ear of corn. Excessive offset angle of the peeling roller will reduce the force between the peeling roller and the ear of corn, which will affect the peeling effect of the bud. The best combination that affects the test index crush rate is A2B1C2D3. The primary and secondary relationships that affect the crush rate are D>A>C>B. The test shows that the offset angle of the peeling roller and the rotation speed of the pulverizing drum are the main factors affecting the qualified rate of pulverization. The offset angle of the peeling roller affects the peeling effect of the bracts. To obtain a high qualified rate of blister crushing, first, the peeling effect of the bracts is better. The torn and layered bract leaves fall into the crushing device, and the crushing effect is poor. The main reason is that the whole bract leaves have a large gravity, have a large contact area with the blade on the crushing drum, and are crushed by a blade that is rotating at a high speed.

**Table 7:** Combination and results of orthogonal test factors.

Experiment number	Experimental factors													Experimental results			
	A	B	A×B	C	A×C	B×C		D	A×D	B×D	C×D			η_1_	η_2_	η_3_	η_4_
	1	2	3	4	5	6	7	8	9	10	11	12	13	%	%	%	%
1	1	1	1	1	1	1	1	1	1	1	1	1	1	0.31	95.69	0.66	89.1
2	1	1	1	1	2	2	2	2	2	2	2	2	2	0.4	96.08	6.0	89
3	1	1	1	1	3	3	3	3	3	3	3	3	3	0.17	94.55	4.0	93
4	1	2	2	2	1	1	1	2	2	2	3	3	3	0.43	96.19	1.6	91
5	1	2	2	2	2	2	2	3	3	3	1	1	1	0.1	93.96	1.16	94.3
6	1	2	2	2	3	3	3	1	1	1	2	2	2	0.53	95.34	0.4	90.7
7	1	3	3	3	1	1	1	3	3	3	2	2	2	0.21	95.16	1.46	95
8	1	3	3	3	2	2	2	1	1	1	3	3	3	0.31	95.49	0.60	88
9	1	3	3	3	3	3	3	2	2	2	1	1	1	0.05	94.87	1.16	90.8
10	2	1	2	3	1	2	3	1	3	3	1	2	3	0.45	95.27	1.02	93.4
11	2	1	2	3	2	3	1	2	1	1	2	3	1	0.17	94.43	0.91	92.8
12	2	1	2	3	3	1	2	3	2	2	3	1	2	0.97	94.84	1.02	96
13	2	2	3	1	1	2	3	2	1	1	3	1	2	0.01	96.29	2.56	93.8
14	2	2	3	1	2	3	1	3	2	2	1	2	3	0	96.03	2.1	96
15	2	2	3	1	3	1	2	1	3	3	2	3	1	0.44	96.92	0.24	88.3
16	2	3	1	2	1	2	3	3	2	2	2	3	1	0	94.15	0.92	96
17	2	3	1	2	2	3	1	1	3	3	3	1	2	0.5	96.75	0.98	92.2
18	2	3	1	2	3	1	2	2	1	1	1	2	3	0.15	97.42	1.07	93.6
19	3	1	3	2	1	3	2	1	2	2	1	3	2	0.52	94.80	1.02	90.1
20	3	1	3	2	2	1	3	2	3	3	2	1	3	0.22	95.17	1.55	95.8
21	3	1	3	2	3	2	1	3	1	1	3	2	1	0	94.22	1.61	97
22	3	2	1	3	1	3	2	2	3	3	3	2	1	0.15	94.99	2.10	96.7
23	3	2	1	3	2	1	3	3	1	1	1	3	2	0.08	93.93	1.73	96
24	3	2	1	3	3	2	1	1	2	2	2	1	3	0.18	95.88	0.89	89.1
25	3	3	2	1	1	3	2	3	1	1	2	1	3	0.06	95.68	2.40	89
26	3	3	2	1	2	1	3	1	2	2	3	2	1	0.27	94.61	1.29	93
27	3	3	2	1	3	2	1	2	3	3	1	3	2	0.16	94.82	2.09	91

**Table 8:** Orthogonal test variance analysis table.

Sources	η_1_			η_2_			η_3_			η_4_		
of variation	Sum of squares	MS	Sig.	Sum of squares	MS	Sig.	Sum of squares	MS	Sig.	Sum of squares	MS	Sig.
A	0.07			3.599			2.191			27.816		
B	0.147	0.035	0.505	1.319	1.8	0.21	2.208	1.096	0.322	4.116	13.908	0.176
A×B	0.125	0.073	0.268	1.231	0.66	0.525	2.907	1.104	0.319	0.934	2.058	0.733
C	0.036	0.063	0.317	1.879	0.616	0.546	8.563	1.454	0.236	22.001	0.467	0.93
A×C	0.023	0.018	0.693	0.346	0.94	0.41	0.851	4.281	0.037	3.256	11	0.239
B×C	0.123	0.012	0.787	0.817	0.173	0.836	2.288	0.426	0.619	2.992	1.628	0.781
D	0.253	0.061	0.323	4.736	0.408	0.663	8.742	1.144	0.308	82.065	1.496	0.796
C×D	0.055	0.127	0.128	0.228	2.368	0.142	0.839	4.371	0.035	12.59	41.033	0.022
error	0.376	0.028	0.578	7.538	0.114	0.888	6.683	0.42	0.623	51.085	6.295	0.414
Total	3.024	0.047		245320.16	0.942		103.242	0.835		231824.31	6.386	

The analysis of variance of the orthogonal test results is shown in Table 8. The analysis results of the damage rate of the test indicators show that the significant values of the interaction terms are all greater than 0.05, and there is no interaction between the factors. The analysis results of the stripping rate variance for the test indicators show that the significant values of each interaction term are greater than 0.05, which indicates that there is no significant effect on the stripping rate and there is no interaction between the factors. The analysis of variance of the loss index for the test indicators shows that the significant values of each interaction term are greater than 0.05, the impact on the loss rate is not significant, and there is no interaction between the factors. The analysis of variance of the crush rate of the test index showed that the significant value of each interaction term was greater than 0.05, the impact on the loss rate was not significant, and there was no interaction between the factors.

From the test data and results, we can also see that there was no interaction between the four factors: the rotation speed of the crushing drum, the distance between the peeling and the axis of the crushing device, the speed of the peeling roller, and the offset angle of the peeling roller. Based on the comprehensive balance test indicators, the optimal parameter combination of a bract leaf peeling and crushing device is obtained: the rotating speed of the crushing drum is A2B3C2D1.

### Multiple corn feeding tests

**Fig 10:**
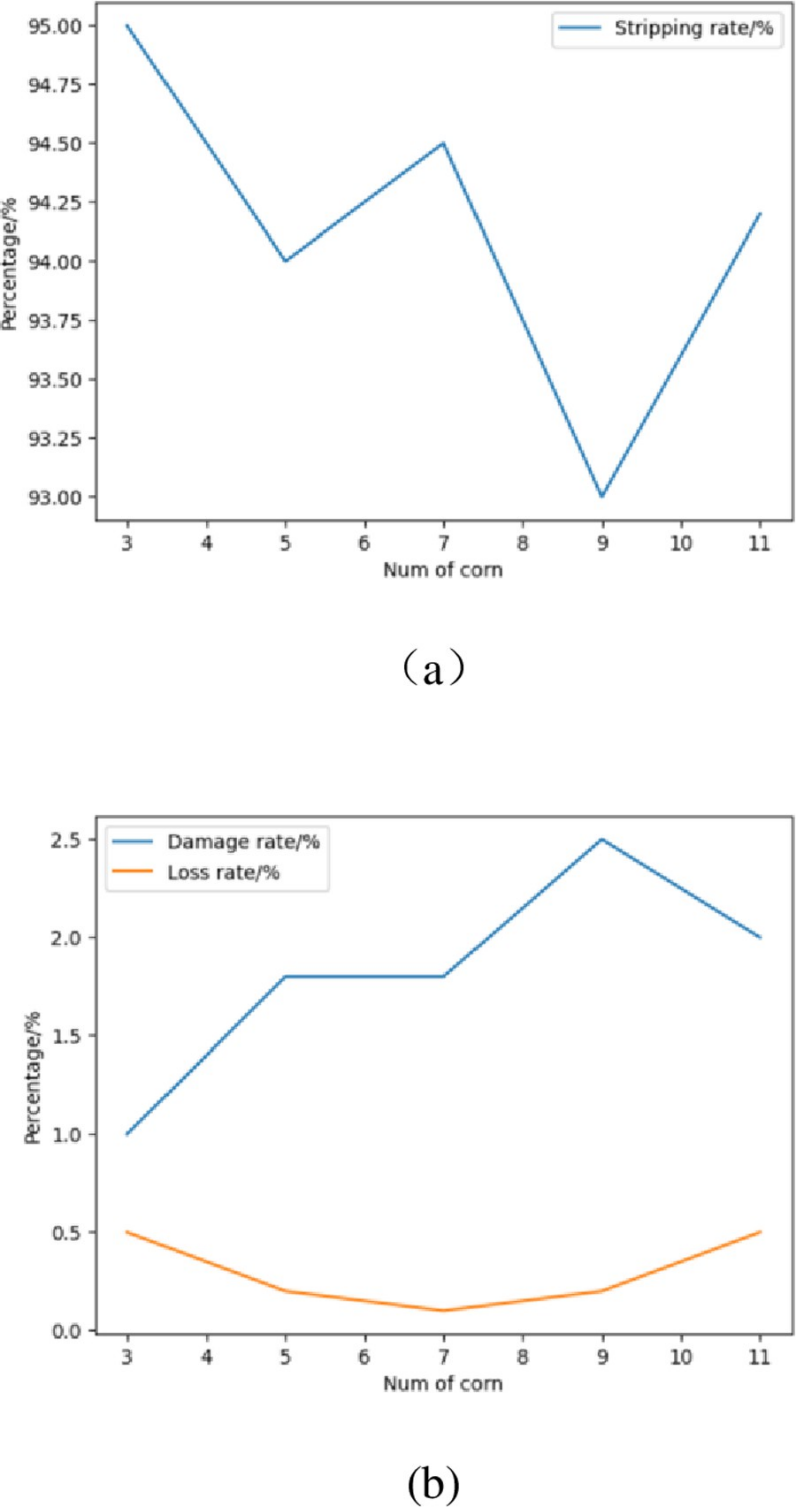
Effect of corn feeding amount on test index.

According to the randomness of the number of ears of corn entering the device, further tests were performed on 3, 5, 7, 9, and 11 ears of corn placed in the device. The test results are shown in Fig 10. According to the line chart, the effect of the amount of corn on the peeling rate, breakage rate, and loss rate is shown. It can be seen in the line graph of experimental data processing that the stripping rate in the three test indicators decreased with the increase in the number of ears of corn, and the breakage and loss rates increased with the increase in the number of ears of corn. According to the actual situation of corn stripping, we require that the larger the stripping rate, the better, and the smaller the damage rate and loss rate, the better. The comprehensive balance test index shows that the effect of the three ears of corn fed at the same time is the best.

According to F inspection tables *F*_0.90_(3,16) = 2.46, *F*_0.95_(3,16) = 3.24 and *F*_0.99_(3,16) = 5.29, It can be obtained from Table 9. When α = 0.01 or α = 0.05, the F value is greater than the critical value of F, which proves that the result is significant. it is concluded that the amount of corn has a significant effect on the loss rate, so the selection of the number is mainly based on the damage rate.

**Table 9:** Variance processing of corn number variable data.

Source of variance		Sum of squared variance	Degrees of freedom	Mean square	F
Quantitative factors	Tripping rate	7.67	3	2.56	3.3
	Loss rate	4.68	3	1.56	7.4
	Damage rate	0.44	3	0.15	1.5
Error	Tripping rate	12.18	16	0.76	
	Loss rate	3.33	16	0.21	
	Damage rate	1.64	16	0.10	

The relationship between the number of crushed leaves of different corn bracts and the qualified rate of crushing is shown in Table 6. It can be seen from the table that with the gradual increase in the number of corn ears, the crushing success rate of corn bracts and leaves gradually decreases. The optimal number of corn ears to smash is 3 or 5. It was observed from the experiment that with the increase in the number of ears of corn, the pulverizing effect of the pulverizing device of the bract leaf gradually weakened, mainly due to the structure of the pulverizing device of the bract leaf and the gravity of the bract leaf itself. The box of the corn bract leaf crushing device is transparent, the upper part is the feeding inlet of the corn bract leaf, and the lower part is the discharge outlet of the corn bract leaf after being crushed by the crushing drum. As the number of corn ears increases, the number of corn bracts also increases. As the number of corn bracts increases, the weight also increases. The increased corn bracts will be crushed from the corn bracts due to gravity. The corn will be discharged from the space of the corn bract leaf crushing device. Therefore, as the corn bract leaves increase, the pulverizing effect of the bract pulverizing device will weaken.

### High-speed photography test

According to the optimal parameter combination obtained by orthogonal test, we set the rotation speed of the peeling roller to 450 r/min for a high-speed photography test. After the rotation was stable, the ears of corn were inserted. When t = 0.118 s, the unpeeled ears were placed in the device at the power input end. At t = 0.145 s, the corn beat and changed its placement direction. Through the rotation friction of the peeling roller, most of the bract leaves were peeled off at t = 0.161 s, and the direction of the ear placement was changed to restore the longitudinal direction. At t = 0.178 s, the corn that was placed in the vertical direction took the remaining bract leaves and peeled them under the rotation of the peeling roller. By t = 0.301 s, the bracts were completely peeled off; at t = 0.337 s, the ears slid to the tail of the device.

High-speed photography experiments showed that the same peeling process at different speeds after the peeling roller changed the rotation speed still could not grasp the corn ear bud leaves placed in the vertical direction. The corn ears were placed horizontally after the beating, and the offset peeling roller easily grabbed most of the bracts of the corn ears in only 0.019s. Then the corn ears with only a few bracts were restored to the vertical placement between the offset peeling rollers. The remaining bracts were easily grabbed, torn, and peeled off. Finally, under the action of the peeling roller, the ear moved to the tail of the device to complete the peeling work.

The results of the high-speed photographic test when the rotating speed of the pulverizing roller of the corn bract pulverizing device was 1,300 r/min. The beginning of the contact between the bract leaves and the pulverizing drum was recorded as t = 0.050s. When the bract leaves came into contact with the pulverizing drum (t = 0.050s), as the pulverizing drum rotated, the bract leaves rotated with the pulverizing drum.The corn bract leaves were first crushed by the grinding moving blade (t = 0.133s), then crushed by the grinding moving blade and the grinding fixed blade (t = 0.150s). Under the action of inertia and torque, the corn bract leaves rotated together with the pulverizing drum, the corn bract leaves were repeatedly pulverized by the pulverizing blade and the pulverizing fixed blade (t = 0.160s), and, finally, the pulverization of the corn bract leaves was completed (t = 0.210s).

The high-speed photography test showed that compared with the speed of other pulverizing rollers, the time to complete the pulverization of the bract leaves was significantly shortened, and the effect of pulverizing the leaves was more ideal. However, because the rotating speed of the pulverizing drum was relatively fast, the phenomenon of bounce and slip of corn bract leaves under the action of torque was also more significant, and the probability of bract leaves falling off the corn bract leaf pulverizing device was also greater.

## Conclusion

(1) The device for peeling and crushing corn bract leaves was designed, and the kinematics and dynamics of the peeling process of corn bract leaves were analyzed. Based on theoretical analysis results, four factors, including the rotation speed of the grinding drum, the distance between the axis of the grinding device and the axis of the peeling device, the speed of the peeling roller, and the offset angle of the peeling roller, were determined for orthogonal experiments. The test results showed that, according to the comprehensive balance test index, the best parameter combination of a bract leaf peeling and pulverizing device is as follows A2B3C2D1.

(2) Based on the optimal parameters, several corn feeding experiments and high-speed photography experiments were performed. The results of multiple corn feeding experiments showed that when three ears of corn were fed at the same time, the effect of the bract leaf peeling and pulverizing device was the best. The high-speed photography test showed that the peeling roller only took 0.019 s to grip most of the bract leaves of the corn ear, and then the remaining few bract leaves were restored to the vertical placement between the peeling rollers so that they could be easily grasped, torn, and peeled. The larger the rotation speed of the pulverizing drum, the better the pulverizing effect of the bract leaves. If the rotation speed was too fast, the phenomenon of skip and slip occurred. In order to prevent the phenomenon of jumping and slipping, the rotating speed of the grinding drum should be controlled within a reasonable range when using the test device for experiments.
